# Editorial: Recent advancements and developments in targeted drug delivery systems for cancer diagnosis and therapy

**DOI:** 10.3389/fddev.2025.1654788

**Published:** 2025-09-10

**Authors:** Abhishesh Kumar Mehata, Bipin Chaurasia, Ana Isabel Fraguas-Sánchez

**Affiliations:** ^1^ Department of Pharmaceutical Engineering and Technology, Indian Institute of Technology (BHU), Varanasi, India; ^2^ Department of Neurosurgery, College of Medical Sciences, Bharatpur, Nepal; ^3^ Department of Pharmaceutics and Food Technology, Complutense University Madrid, Madrid, Spain

**Keywords:** drug delivery, nanomedicine, theranostics, cancer therapy, targeted therapy

This Research Topic combines original research and reviews addressing recent strategies in targeted drug delivery systems for cancer diagnosis and therapy. The included studies highlight clinical and preclinical advances, from chemotherapy optimization and immunotherapy evaluation to nanotechnology-based approaches and theranostics. Below, we summarize the key contributions. In a study Tafenzi et al., performed the retrospective study for evaluating second line chemotherapy efficacy in relapsed non-small cells lung cancer (NSCLC) and small cells lung cancer (SCLC) following platinum based treatment failure. There was not any significant improvement in the NSCLC treatment was observed, whereas overall survival was improved in SCLC treated patients. In another study Wu et al., evaluated the cost effectiveness of atezolizumab and chemotherapeutic agent in advanced NSCLC patients, which were not eligible for platinum based treatment. It was reported that atezolizumab offered additional 0.35 quality adjusted life years (QALYs) but exceeded the cost effectiveness thresholds in both the US and China. Similarly Zhang et al., observed that teslizumab offered improved survival benefits over docetaxel in NSCLC with 0.58 QALYs. Recently, Zhou et al., presented the bibliometric and visualization analysis of nanoparticles for gynaecological cancer over past 2 decades. It was noted that China leads in the nanoparticles-based drug delivery publications, whereas silver, gold, nanoparticles and green synthesis are emerging research trends. This systemic review offers the guidance for advancing preclinical to clinical applications. Furthermore, Tanaka et al., demonstrated multicentre retrospective study that evaluated the prognostic values of total baseline tumour size (BTS) in advanced SCLC treated patients with PD-L1 and chemotherapeutic drug. It was observed that patients with large BTS had significantly reduced the survival compared to that of small BTS. Hence, total BTS offers valuable prognostic marker in the first line SCLC immunotherapy.

The utilization of nanomaterials for cancer treatment has attracted substantial interest in the last few decades. Tumour-targeting nanomaterials is a developing discipline with vast implications. It delivers a unique method and complete technologies against cancer through individualized therapy. Target-specific medication treatment and techniques for prompt identification of diseases are the primary study fields in which nanomaterials would serve an important role. Rajapaksha et al., reviewed the emerging theranostic approaches in ovarian cancer that focus the diagnostic and therapeutic aspects of the nanoparticles. The review also focused on the specific genomic and proteomic biomarkers for the detection and treatment of cancer. Overall, the Research Topic Collection reflects the breadth of current innovation—from clinical evidence supporting treatment decision-making to nanotechnology-driven theranostic platforms with potential to transform cancer care.

The editors express deep gratitude to the authors for their significant contributions, the reviewers for their insightful critiques, and the editorial team of Frontiers in Drug Delivery for their professional assistance.

